# A General Super-Resolution Approach Integrating Physical Information for Temperature Field Measurement

**DOI:** 10.3390/s24237445

**Published:** 2024-11-22

**Authors:** Sheng Chen, Zhixuan Su, Min Dai, Chenyang Xue, Jiping Tao, Zhenyin Hai

**Affiliations:** School of Aerospace Engineering, Xiamen University, Xiamen 361102, China; 23220221151741@stu.xmu.edu.cn (S.C.); suzhixuan@stu.xmu.edu.cn (Z.S.); daimin@stu.xmu.edu.cn (M.D.); xuechenyang@xmu.edu.cn (C.X.)

**Keywords:** deep learning, super-resolution, measurement techniques, temperature field

## Abstract

In industrial measurement, temperature field measurement typically relies on thermocouples and spectroscopic techniques. These traditional methods often suffer from insufficient precision, resulting in prevalent low-resolution measurements in real thermal scenarios. To address this challenge, we propose a novel general super-resolution approach for temperature field measurement in various thermal scenarios, leveraging the low-resolution (LR) data obtained from sensor array technology. The method incorporates skip connections and multi-path learning, along with physical information loss, to enhance accuracy. To validate the effectiveness of the approach, simulations across three two-dimensional thermal scenarios are conducted: the heating process in silicon chips, the thermodynamic process of hot and cold water mixing, and the convective heat transfer phenomena involved in metal sheet dissipation under airflow. The results show that the learning model can accurately predict the HR temperature. The proposed approach offers a pathway for generating HR solutions, bypassing traditional time-consuming simulation processes while ensuring data accuracy. By utilizing a fixed model and a lightweight physical loss function, we simplify the deployment process, facilitating applications in computational fluid dynamics (CFD) solutions, engineering measurements, and related fields.

## 1. Introduction

Temperature measurement is central to thermal analysis and condition monitoring in industries such as electronics, pipelines, and aerospace [1,2,3]. By monitoring temperature and related information within thermal fields, safety incidents can be effectively mitigated, thereby improving the safety and reliability of industrial equipment. Furthermore, analyzing temperature data enables a deeper understanding of thermal characteristics, an assessment of heat dissipation under varying operating conditions, and the prediction of potential thermal issues. This ensures more stable and reliable operation throughout the equipment’s service life. Additionally, temperature measurement helps engineers optimize the selection of thermal dissipation structures and materials, enhancing efficiency and performance while reducing energy consumption and costs.

In recent studies, thermal field measurement methods are primarily classified into invasive and non-invasive measurements [4]. Invasive measurements mainly rely on thermocouple technology, while non-invasive measurements primarily use spectroscopic techniques. In recent years, with the advancement of sensor array technology, low-resolution (LR) temperature field data can be obtained at a relatively low cost. Nonetheless, electrical signal interference among adjacent sensors limits the acquisition of accurate and high-resolution (HR) thermal field information. Spectral intensity and environmental factors also pose challenges to applying spectroscopic techniques for obtaining high-resolution data at a low cost [5,6]. Therefore, there is an urgent need for effective and low-cost measurement approaches to obtain high-resolution temperature data across various thermal fields.

To address this challenge, some pioneers have proposed combining LR data with deep learning techniques to develop super-resolution methods. These methods aim to use machine learning models to learn physical field distributions and capture physical characteristics. Deep learning has been widely recognized for its powerful search and computational capabilities, enabling many innovative applications across diverse fields [7]. Super-resolution (SR) is a technique for enhancing image resolution. Deep learning-based methods for SR were initially introduced by Dong et al. [8]. They proposed a deep learning model, the super-resolution convolutional neural network (SRCNN), which exhibits significantly superior SR reconstruction performance compared to traditional methods such as bicubic interpolation [9]. As one of the earliest deep learning approaches for super-resolution, this technique has been extensively validated in fields such as medicine [10], fluid mechanics, and beyond. The related research in thermodynamics [11] is also increasing year by year, and it has become a reliable choice for the measurement and study of thermal phenomena. SRCNN is capable of reconstructing fine details and complex structures in temperature fields, which is essential for accurate analysis in thermodynamic applications. This capability arises from deep learning models’ ability to capture complex patterns often overlooked by traditional methods.

Building on SRCNN, researchers have proposed increasingly complex models to handle diverse applications. Kong et al. [12] explored deep learning-based approaches in heat flow fields, proposing a novel multiple-path super-resolution convolutional neural network (MPSRC) for temperature measurement in combustion chambers. Heuristically, we realize that the flow and temperature fields in the SR task exhibit similar structural and spatial dependencies. In this paper, we draw on mainstream studies in flow field super-resolution to inform our approach. Fukami et al. [13] applied SR techniques in fluid dynamics, proposing a hybrid downsampled skip-connection/multi-scale (DSC/MS) model for reconstructing two-dimensional decaying isotropic turbulence flow fields. Deng et al. [14] introduced two Generative Adversarial Network (GAN)-based models, Super-Resolution Generative Adversarial Network (SRGANs) and Enhanced Super-Resolution Generative Adversarial Networks (ESRGANs), for booting the spatial resolution of complex wake flows behind cylinders.

With the advancement of SR algorithms, approaches relying solely on SRCNN have been considered as being limited in interpretability. To address this, some researchers have incorporated physics-informed neural networks (PINNs) [15,16,17] and developed methods to integrate physical information during training, enhancing both interpretability and accuracy [18,19]. Rajat et al. [20] introduced specific physical losses during training to enhance discontinuity detection in applications such as Burger’s equation, methane combustion, and fouling in industrial heat exchangers. Building on the widely used U-Net architecture for image segmentation, they developed PI-UNet, a tailored deep learning model for these tasks. In our research, three different scenarios are also selected to validate the effectiveness of our model. To provide a benchmark for comparison, we utilized the PI-UNet, that has demonstrated strong performance across various scenarios.

Drawing inspiration from existing studies and incorporating physical information, we propose a modified model based on the previously mentioned DSC/MS framework. This model has been further optimized from DSC/MS for application in temperature field environments. The key rationale behind choosing the DSC/MS model lies in its dual-path architecture, which enables it to independently learn the characteristics of the temperature field at various scales. This design facilitates the precise capture of smooth gradient properties and sparse mutations within the temperature field, enhancing the model’s expressiveness and overall applicability. Inspired by PI-UNet, we introduce a simplified loss function that integrates physical information loss, further enhancing the model’s accuracy. Additionally, the simplified design mitigates the loss of physical information through automatic differential back propagation and the preservation of differential matrices, resulting in reduced computational costs. We conduct simulations across three distinct two-dimensional thermal scenarios to evaluate the effectiveness of our approach: laser heating dynamics within silicon chips, the thermodynamic interaction of merging hot and cold water, and the convective heat transfer phenomenon associated with heat dissipation in metal sheets exposed to airflow. The main contributions of this research are as follows:

- Propose the modified DSC/MS model to effectively address the SR task in temperature fields.
- Introduce simplified physical loss, which incorporates physical information into guided training while reducing the full calculation cost from physical information loss.

The remainder of this paper is organized as follows: Section 2 introduces the datasets used in this study. Section 3 describes the architecture of the super-resolution model and the specific physical loss function. In Section 4, we showcase the SR reconstruction performance of our proposed model on three scenarios. We also highlight the positive effects of physical information on training. Finally, Section 5 provides a summary.

## 2. Dataset

The primary objective of this study is to design a general supervised model F:LR→HR that effectively captures the nonlinear relationship between LR data in a heat flow field and HR data, where $(x=x_{1},x_{2},\ldots ,x_{n})\in \mathrm{LR}$ represents the LR data and $(y=y_{1},y_{2},\ldots ,y_{m})\in \mathrm{HR}$ represents the HR data with n≪m. We apply this model to the following three different thermal fields:

- Laser Heating: A silicon wafer with a 2-inch diameter and a notch on the left side is fixed on a thermally insulated workbench inside a chamber. The workbench rotates at 10 revolutions per minute. The wafer is heated by two laser beams, each with a radius of 0.01 m and a power of 10 W. The lasers move along the *x* and *y* axes of the silicon wafer. The heating process lasts one minute. In this experiment, it is assumed that the thermal insulation of the environment is good and the chamber walls maintain a constant temperature of 20 °C. Under these conditions, the only source of heat loss is thermal radiation transmitted from the top of the wafer to the chamber walls.
- Water Mixing: In a container measuring 0.03 m × 0.1 m, filled with cold water at ambient temperature, hot water at 90 °C is injected through an upper channel with a diameter of 30 mm at a velocity of 0.2 m/s. The mixed water flows out from the bottom, forming a temperature field. In the simulation process, the effect of gravitational convection on the flow is neglected.
- Heat Dissipating: The bimetallic strip, fixed on the left side inside an air duct, is heated by a constant 50 W heat source to induce bending. Airflow is introduced to cool the metal plates. The geometric structure of the bimetallic strip consists of two plates, each measuring 0.01 m × 0.05 m, with the lower one made of copper and the upper plate made of iron. An airflow at a speed of 0.2 m/s flows from the left side of the channel to the right side.

**Figure 1:**
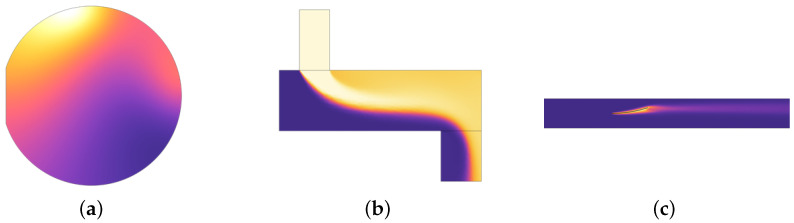
Schematic diagrams of (**a**) laser heating scenario, (**b**) water mixing scenario, and (**c**) heat dissipation scenario obtained from COMSOL simulation.

These three simulation cases are sourced from the COMSOL Multiphysics official case library referred to as laser heating [21], water mixing [22], and heat dissipating [23], respectively. The simulation difficulty of the three scenarios increases gradually, corresponding to an increment in the number of partial differential equations to be considered. We simulate these thermal fields using COMSOL Multiphysics 6.2, obtaining a certain quantity of HR data at various time steps. The schematic diagram of the simulation scenarios is shown in Figure 1.

The lighter shades indicate regions of lower temperature, while warmer tones represent areas of higher temperature.

The simulation data can be considered as accurate high-resolution data, which are post-processed into temperature field images of a size of 128×128. To align with the sampling principles of spectral and sensor array technologies for low-resolution data, we apply average pooling to the high-resolution images to obtain LR data. Average pooling is a method used to compress input feature maps by reducing dimension. To transform a high-resolution image of size α×β into a low-resolution grid of size α/M×β/M, we represent the average pooling operation as

(1) $$q_{ij}^{\mathrm{LR}}=\frac{1}{M^{2}}\sum_{n,m\in P_{\left(i,j\right)}}q_{nm}^{\mathrm{HR}},$$

where $q_{ij}^{\mathrm{LR}}$ denotes the values in the coarse mesh, $q_{nm}^{\mathrm{HR}}$ denotes the values in the fine mesh, and $P_{\left(i,j\right)}$ denotes a range in which the HR values are averaged to obtain the corresponding $q_{ij}$. Since physical measurement sensors typically consider the LR data as the average temperature within a region, applying average pooling to process the data is reasonable. Additionally, referring to Fukami’s work [13] on image processing for fluid dynamics, average pooling shows superior performance in super-resolution prediction compared to max pooling.

We sampled 2100 data points at a regular interval from the beginning of the simulation. The samples are divided into a training set of the first 2000 points and a test set of the remaining 100 points. The specific simulation and sampling parameters are shown in Table 1.

## 3. Method

### 3.1. Model Structure

**Figure 2:**
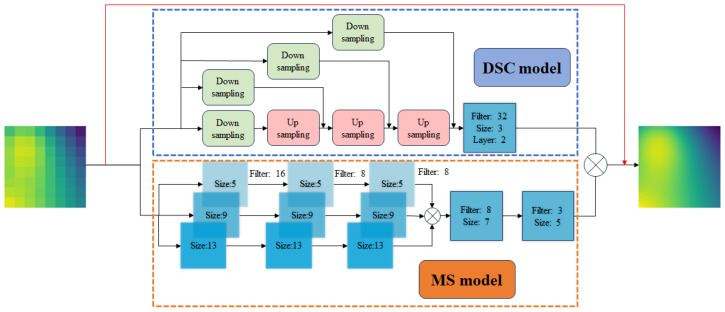
The improved DSC/MS model is the DSC model in the blue dashed line box at the top and the MS model in the orange dashed line box at the bottom.

We consider using a classical super-resolution model named DSC/MS [13], as depicted in Figure 2. The model combines a hybrid downsampled skip-connection (DSC) model and a multi-scale (MS) model to capture both large and small structures. The DSC model extends the CNN model by introducing compression and skipping connections, as shown in the path within the dashed blue box in Figure 2. In super-resolution analysis, data compression (downsampling) and restoration (upsampling) enhance the robustness of data element translation and rotation [24]. The downsampling sampling rates, not mentioned in the figure, are 8, 4, and 2 from left to right. The upsampling sampling rate is 2. Skip connections [25] enable the network to directly access detailed information from lower layers and propagate it to higher layers, effectively addressing issues of gradient vanishing and information loss. Unlike the DSC model, which focuses on large-scale structures, the MS model proposed by Du et al. [26] captures small-scale structures in the data. Small-scale structures typically refer to local variations and minor features in the temperature field, such as hot spots and thermal gradients. The MS model is composed of multiple CNN filters with different sizes. By focusing on small structural features of different scales simultaneously, the model’s flexibility increases, thus improving its generalization ability and robustness. As shown in the path within the orange dotted box in Figure 2, the CNN filter is represented by a blue box. The number after “filter” denotes the number of output channels, the number after “Size” denotes the convolutional kernel size, and “Layer” denotes the number of repeats of the CNN filter of the same size. If “Layer” is not marked, the default value is 1. Similar to Figure 1, areas close to blue are areas of low temperature and areas close to yellow are areas of high temperature.

Given the relatively fewer abrupt changes and smoother gradient characteristics in the temperature field, we make some modifications to the original DCS/MS model by adding a skip connection just before the final output layer. The added skip connection allows the original low-resolution data to directly participate in the predictions of the last layer of the network. The rationale behind this modification lies in the intrinsic characteristics of the temperature field, where the LR grid inherently embodies certain distributional information. However, during network propagation, some information is inevitably lost. We augment the influence of the LR grid in the final calculation layer by incorporating a skip connection. This enhancement facilitates a more robust capture of the temperature field distribution.

### 3.2. Loss Function

The loss function serves as the backbone of the learning process. By minimizing the loss function, the neural network adjusts its hyperparameters to better fit the known data. This process also enhances its ability to generalize to unknown data. Considering the significance of the loss function, we propose the introduction of a physics-based loss function that integrates both supervised data loss and physical loss components. This approach is widely used when addressing problems governed by known physical equations. A pioneering work is the PINNs introduced in [15]. In PINNs, the loss function includes terms related to both supervised data fitting and the residual of conservation laws. The residual terms typically include residuals of initial and boundary conditions, and the residual of partial differential equations at selected points in the time–space domain. Specifically, we propose using the partial differential equation controlling the physical process, as follows:

(2) $$u_{t}+N\left[u;\lambda \right]=0,\;x\in \Omega ,\;t\in \left[0,T\right].$$

In Equation (2), *x* denotes a spatial variable and Ω denotes a spatial domain. *t* denotes the time variable, and *T* is the simulation period. *u* denotes an unknown function with respect to *x* and *t* to be solved, which typically represents temperature, velocity, or other physical quantities of interest depending on the problem. $u_{t}$ denotes the partial derivative of the function *u* with respect to time *t*. *N* denotes a linear or nonlinear operator that acts on a function *u*, which is often some kind of differential operator, like the gradient or divergence. λ is an additional parameter or auxiliary variable. We define $\hat{u}$ as the predicted result. The physical loss $Loss_{f}$ can be formulated as

(3) $$Loss_{f}=\frac{1}{N_{f}}\sum_{i=1}^{N_{f}}{\left({\hat{u}}_{t}+N\left[\hat{u};\lambda \right]\right)}^{2},$$

where $N_{f}$ is the number of predicted results, which in this case is the number of HR points. Customized loss functions like Equation (3) are often employed alongside the data loss $Loss_{d}$ to guide model training:

(4) $$Loss_{d}=\mathrm{MSE}\left(u,\hat{u}\right).$$

We have studied the effects of various physical quantities in the thermal scenario, and the main equation related to temperature, the heat conduction equation, is described as

(5) $$\rho C_{p}\left(\frac{\partial T}{\partial t}+\left(u\cdot \nabla \right)T\right)=\nabla \cdot \left(k\nabla T\right)+Q,$$

where ρ, $C_{p}$, and *k* are scenario parameters, representing density, isobaric specific heat capacity, and thermal conductivity of the heat transfer medium. *Q* denotes the heat source term and *u* is the fluid velocity. The gradient operator is denoted by ∇. Equation (5) describes heat transfer within the fluid. If there is no fluid flow, the convective term u·∇T is zero.

To construct the physical loss function (i.e., Equation (3)), we must model the heat transfer environment in detail. This requires considering Equation (5) and other boundary conditions, such as periodic or radiative boundary conditions. Real-world modeling requires considering interactions and effects of different mechanisms. Additionally, model validation demands large datasets, which can be costly when applying Equation (3). On the other hand, calculating physical losses requires solving multi-order partial differentials. If automatic differentiation based on backpropagation is used, the calculation graph becomes too large when working with image outputs of size 128×128. This results in low calculation efficiency and high memory usage. The use of numerical differentiation can help to reduce some computational costs. However, due to the large resolution, it is still necessary to store five first-order and second-order bias matrices of size 128×128, which results in significant memory usage. To address these issues, we aim to develop a simplified physical loss function, leveraging convenient dataset processing to reduce both sampling and computation costs.

Equation (5) describes heat propagation in temperature gradient space through the diffusion term ∇·(k∇T). This term is used in all heat conduction equations, including those for thermal expansion and heat and mass transfer. We utilize an HR temperature distribution as the data label, so that current HR data can be calculated directly to estimate diffusion terms:

(6) $$\begin{array}{cc} \nabla \cdot (\nabla T) & =\frac{\partial^{2}T}{\partial x^{2}}+\frac{\partial^{2}T}{\partial y^{2}}\\ \; & \approx \frac{T_{i+1,j}-2T_{i,j}+T_{i-1,j}}{\Delta x^{2}}\\ \; & \;+\frac{T_{i,j+1}-2T_{i,j}+T_{i,j-1}}{\Delta y^{2}}. \end{array}$$

Therefore, we consider using the diffusion term directly as a physical constraint for supervised learning. Based on the description above, we choose to develop a redesigned loss function to simplify the construction of the physical loss:

(7) $$Loss_{f}=L1\left(\nabla \cdot \nabla T,\nabla \cdot \nabla \hat{T}\right),$$

(8) $$Loss=Loss_{d}+\alpha Loss_{f},$$

where $\hat{T}$ denotes the predicted temperature. We adjust the weights α in the loss expression to adapt to different physical scenarios in datasets. In the subsequent experiments in Section 4, we will demonstrate the validity and generality of this construction approach. Specifically, we will show that diffusion terms can independently accelerate training and improve model performance.

## 4. Results and Discussion

In this section, we present and discuss experimental results, showing the performance evaluation of our proposed method on multiple datasets. In Section 4.1, we introduce the training environment and parameter settings. Two widely used interpolation techniques, bilinear interpolation (BI) and bicubic interpolation (BC), serve as baseline methods in Section 4.2. We demonstrate the impact of our modified DSC/MS model in comparison to the original DSC/MS and PI-UNet [20] model, both reproduced for a fair comparison. In Section 4.3, we show how our proposed simple physical loss improves predictive outcomes, abbreviating our improved models as modified DSC/MS and modified PI-DSC/MS, where “PI” indicates that the model incorporates physical information in the loss function. Finally, in Section 4.4, we compare the effect of sample size on model training effectiveness.

### 4.1. Model Training

Model training is conducted on a server equipped with an RTX4090 GPU (NVIDIA Corporation, Santa Clara, CA, USA), 32 GB memory, and 8 CPU cores. The open-source software library PyTorch 2.1.2 is used. We fix the number of training iterations at 2000 and the sample size at 2000 for all three simulations. We use the Adam optimizer with an initial learning rate of 0.001 and a batch size of 100. To prevent overfitting, L2 regularization (weight decay) is applied with a coefficient of 0.00001. Also, gradient clipping is used to prevent gradient explosion. The model with the best test loss during iteration is saved as the final model.

### 4.2. Super-Resolution Results

To evaluate model performance, we selected four comparison metrics: mean absolute percentage error (MAPE), L2 Error, peak signal-to-noise ratio (PSNR), and structure similarity index measure (SSIM). The calculation methods are as follows:

(9) $$\mathrm{MAPE}=\frac{1}{n}\sum_{i=1}^{n}\left|\frac{y_{i}-{\hat{y}}_{i}}{y_{i}}\right|\times 100\%,$$

(10) $$L2\;\mathrm{Error}=\frac{1}{n}\sqrt{\frac{\sum_{i=1}^{n}{\left(y_{i}-{\hat{y}}_{i}\right)}^{2}}{\sum_{i=1}^{n}{\left(y_{i}\right)}^{2}}},$$

(11) $$\mathrm{PSNR}=10\cdot \log_{10}\left(\frac{{\mathrm{MAX}}^{2}}{\mathrm{MSE}}\right),$$

(12) $$\mathrm{SSIM}=\frac{\left(2\mu_{y}\mu_{\hat{y}}+C_{1}\right)\left(2\sigma_{y\hat{y}}+C_{2}\right)}{\left(\mu_{y}^{2}+\mu_{\hat{y}}^{2}+C_{1}\right)\left(\sigma_{y}^{2}+\sigma_{\hat{y}}^{2}+C_{2}\right)}.$$

In the above formulas, *n* denotes total quantity, *y* represents ground truth, and $\hat{y}$ denotes predicted value. MAX refers to the maximum predicted value and MSE stands for the mean squared error. μ represents the local mean, $\sigma^{2}$ denotes the local variance, and $\mu_{y}\mu_{\hat{y}}$ represents the local covariance. These local features are obtained by the convolution operation of the sliding window, and the kernel size of the window is 11. $C_{1}$ and $C_{2}$ are small constant values introduced to stabilize the computation and avoid division by zero in the denominator. MAPE visually measures average pixel-level differences between two images but ignores the intensity of the differences. The L2 Error indicates how similar a predicted image is to the original, being sensitive to noise and potentially not matching a human eye perception of image quality. PSNR represents the ratio of dynamic range of original image to noise in a reconstructed image, evaluating the quality of the reconstructed image. Larger PSNR values indicate less image distortion. Generally, PSNR above 40 dB indicates an image quality that is nearly indistinguishable from the original. A range between 30 and 40 dB indicates distortion loss within an acceptable range. An image quality below 30 dB is considered poor. In the following experiments, when PSNR is below 20 dB, we believe that the SR method has no effect. SSIM is a more perceptually meaningful metric for assessing image quality, as it focuses on structural information rather than simply pixel-level differences. SSIM offers a more comprehensive evaluation of image quality, providing a better reflection of human visual perception.

**Figure 3:**
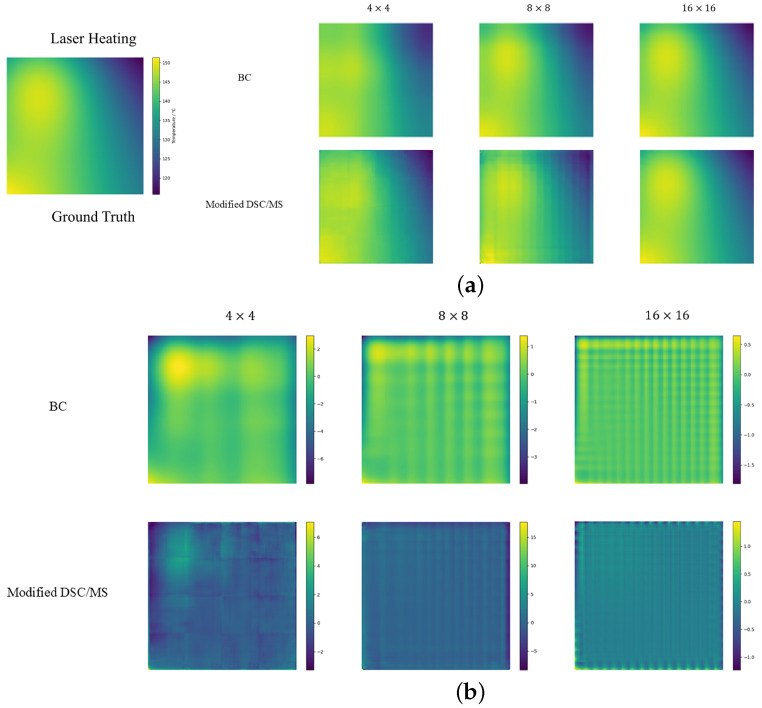
Schematic diagrams of (**a**) prediction and (**b**) error for laser heating scenario.

**Figure 4:**
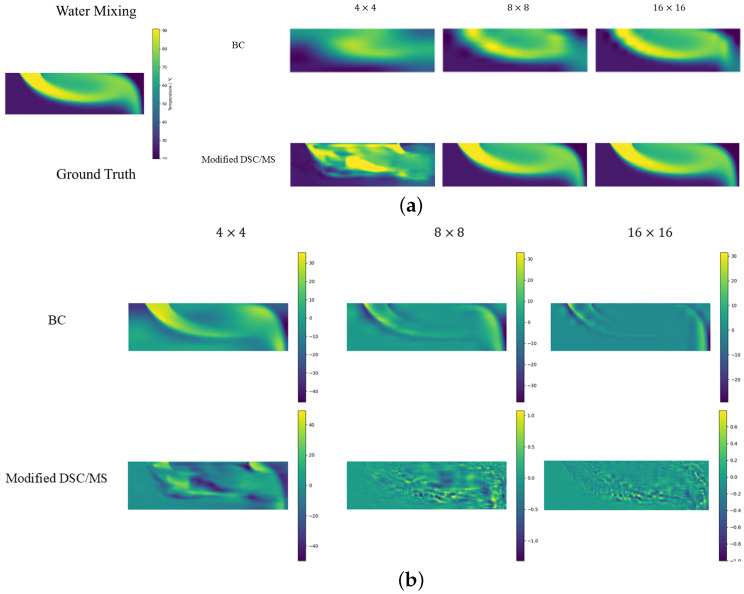
Schematic diagrams of (**a**) prediction and (**b**) error for water mixing scenario.

**Figure 5:**
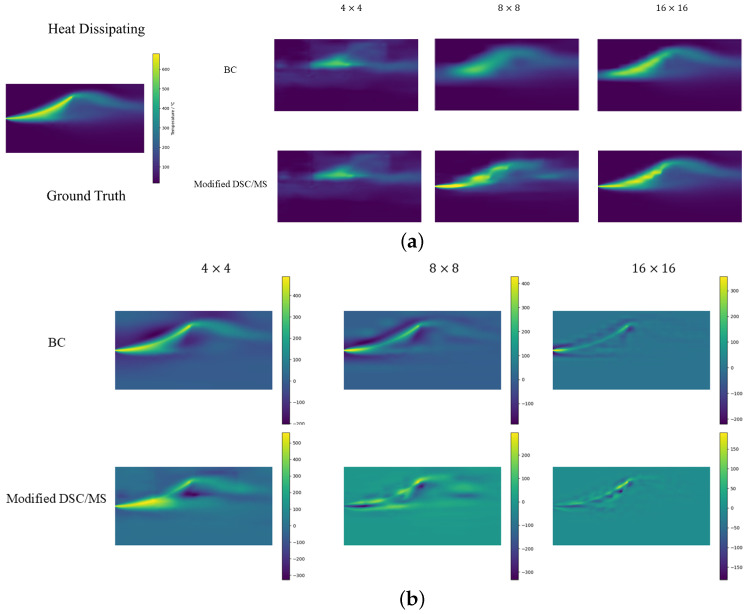
Schematic diagrams of (**a**) prediction and (**b**) error for heat dissipating scenario.

In Section 4, we present statistics for different forecast results in Table 2, Table 3 and Table 4. In Figure 3, Figure 4 and Figure 5, results for SR in three scenes are shown, along with error graphs (using better BC and improved DSC/MS model examples).

#### 4.2.1. Laser Heating

Table 2 shows that the modified DSC/MS algorithm significantly outperforms other methods in terms of MAPE and L2 Error. While our model performs well in terms of accuracy, its PSNR is lower than that of interpolation methods. Super-resolution models typically produce finer details than interpolation methods, but these details may contain higher frequency noise or artifacts that, while having less of an impact on the error indicator, can cause PSNR to decrease because PSNR is sensitive to high-frequency noise or small fluctuations. Interpolation methods, such as bilinear or bicubic, typically produce smoother images with higher PSNR because they have less noise and more uniform error distribution. However, this smoothing can sacrifice image detail, resulting in poor accuracy metrics like relative error and L2 Error. Although our method performs poorly compared to interpolation methods in terms of PSNR, it is also above 30 dB, which can be considered good performance. For the SSIM metric, the modified DSC/MS model performs slightly worse than the BC and BI methods, but it still remains within the range of structural similarity. From the error image in Figure 3b, our model’s error interval is similar to the BC method but with a much smoother error distribution and mostly close to zero errors. Therefore, in summary, the prediction results of the modified DSC/MS model still have certain advantages in accuracy. In contrast, the PI-UNet model struggles more with this metric.

#### 4.2.2. Water Mixing

In the convective environment of hot and cold water, our model outperforms other super-resolution strategies on all metrics except for 4×4 LR grids. Modified DSC/MS exhibits significantly lower MAPE and L2 Errors than other methods. The temperature field possesses distinct features in the current environment, such as abrupt changes. The modified DSC/MS model can easily capture these features and make accurate predictions. For the case of an LR grid size of 4×4, all the methods largely fail to produce effective predictions due to the limited feature information provided by the rough grid. As PI-UNet performs better than other models in the case of a 4×4 LR, it can largely be attributed to the positive effect of physical loss in model training. This will be discussed in detail in Section 4.3. Building on this premise, even without employing PI-related methods, our model outperforms PI-UNet in scenarios with LR grids of 8×8 and 16×16, further highlighting the superiority of the DCS/MS structure.

#### 4.2.3. Heat Dissipating

In the heat dissipating scenario, due to temperature aggregation and the large variation in gradients, spatial features are concentrated, posing challenges for super-resolution. With an LR grid size of 4×4, all the methods produce meaningless predictions. Interpolation performs poorly in this scenario. Observing the error graph in Figure 5b, the BC error trends to closely follow the real image, which is also reflected in the SSIM results. Since the interpolation method estimates the surrounding known data points, it may struggle to accurately capture rapid temperature changes in areas with large gradients. This can lead to an overall underestimation of the post-SR temperatures, resulting in lower temperatures after super-resolution and, consequently, a higher MAPE. With an LR size of 8×8, the error remains high. However, as shown in Figure 5a, our proposed model exhibits consistency with the original temperature field in temperature distribution, gradients, and other aspects. With a grid size of 16×16, our model predicts with overwhelming superiority, achieving a 7% higher accuracy than PI-UNet.

Through performance statistics across the aforementioned three environments, we validate the design of our model. The model takes into account features of different scales, enabling it to effectively reconstruct temperature field data at certain scaling ratios. Compared to interpolation methods, the general model exhibits smaller super-resolution errors in scenarios with prominent features and large temperature gradients. Our general model performs significantly better when the LR sizes are 8×8 and 16×16. When the coarse mesh size is 4×4, the model may not be able to complete the super-resolution task in some scenes due to less information. It can also be observed that the improvement of DSC/MS has a certain effect. The modified DSC/MS presents certain advantages in different predictable scenarios, and the effect is significant in some cases. Compared to the interpolation method, although our model is slightly inferior to the interpolation method in some indicators, by looking at the error graph, our model has less noise and less error on the error graph. In contrast, while PI-UNet shows decent predictive performance in specific scenarios, it may exhibit some limitations when performing super-resolution tasks for temperature fields compared to the improved DSC/MS.

### 4.3. Modified PI-DSC/MS

**Figure 6:**
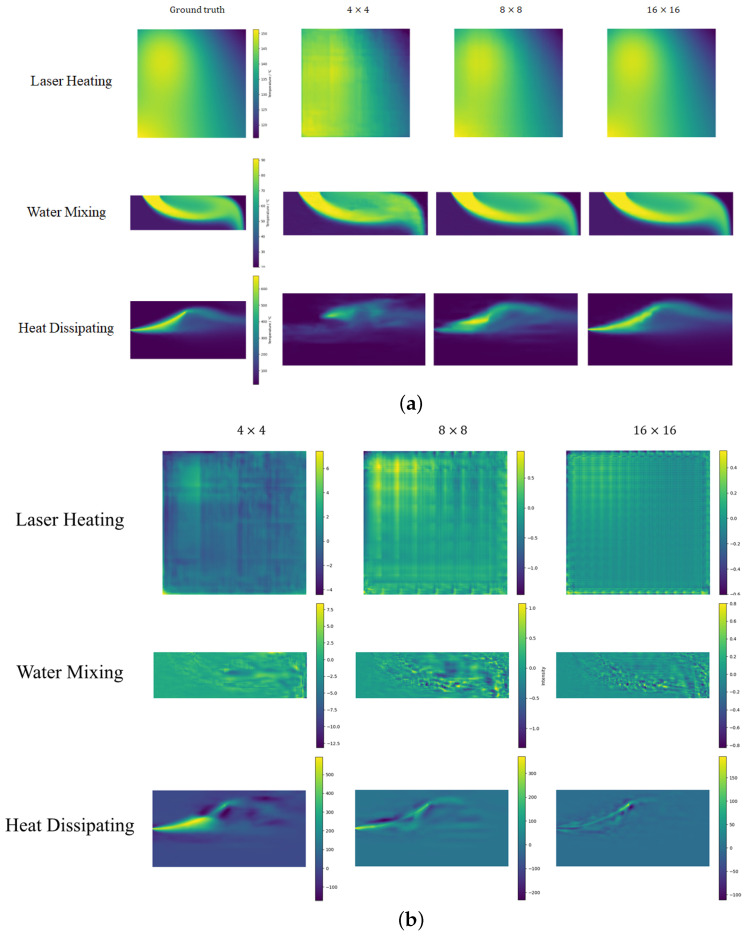
Schematic diagrams of (**a**) prediction and (**b**) error for the scenario from the modified PI-DSC/MS model.

In this section, we consider incorporating a model with specific physical loss functions. Figure 6 and Table 5 demonstrates the impact of the presence or absence of physical loss on prediction performance in three scenarios.

**Figure 7:**
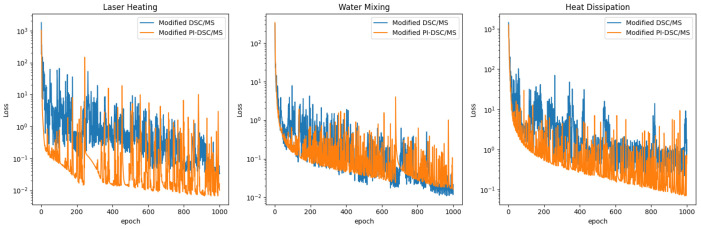
The data loss $Loss_{d}$ of the two SR strategies changes with the training epochs in three scenarios.

Introducing physical loss results in further reductions in predicted MAE and L2 loss, as well as an improvement in PSNR. However, SSIM did not show significant improvement in most cases, suggesting that the modified DSC/MS method is already effective in restoring the temperature field structure. The additional physical residual terms in the loss function serve a similar role as soft constraints on the model, thereby constraining the search space during model training and accelerating convergence. Figure 7 shows the decreasing trend of data loss $Loss_{d}$ in three scenarios. It can be seen that $Loss_{d}$ decreases faster when trained with physical loss constraints. This also highlights the advantage of PI-UNet in the 4×4 low-resolution task within the water mixing scenario, as demonstrated in Table 3. The results demonstrate that the improved PI-DSC/MS not only successfully completes the task but also outperforms PI-UNet across all performance metrics, further confirming the superiority of the structure. It is important to highlight that neither of our models can perform SR in the heat dissipation scenario with a coarse 4×4 grid. As shown in Table 4, none of the five evaluated methods achieved this, indicating that the task is too complex and the 4×4 LR data are insufficient.

### 4.4. Sampling Quantity

**Figure 8:**
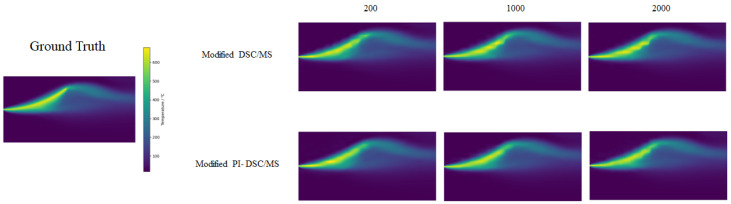
Our SR strategies compare the predictive performance of different sample sizes (200, 1000, and 2000).

As a supplement, we investigated the impact of sampling quantity on the prediction performance of the model. In this section, we conducted experiments using a heat dissipating experiment with an LR size of 16×16 as an example. The results are shown in Table 6 and Figure 8. Although not listed, the predicted effect in the other two scenarios is consistent with the trend of the results in the table.

It can be observed that in most cases, the quantity of samples has minimal impact on the training of the model. Even with a significant reduction in sample quantity, there is no sharp decrease in prediction accuracy. This implies that we can save costs on initial sampling and shorten training time. This feature opens up the possibility for application in scenarios with data distortion caused by long-time sampling of measuring equipment. Based on these findings, we chose to terminate training after 2000 iterations on the grounds that additional iterations would yield diminishing returns in terms of performance improvement. This decision was made to ensure the validity of the model while ensuring the efficiency of the calculation. As a result, as shown in Figure 7, losses continue to decrease, reflecting a continuous but negligible improvement beyond the selected stop point.

## 5. Conclusions

In this paper, we propose a general model for measuring high-resolution temperature fields. We utilize a modified DSC/MS model, which includes a DSC component that effectively captures large-scale features and reflects underlying information, and an MS structure that excels at capturing small features at different scales. Based on the original model, we introduced important modifications such as additional jump joins and specific loss functions. By training and testing the model on numerical simulation temperature fields in three different scenarios, we verify the performance of the modified models. The error of the improved model is consistently below 20% across various scenarios, meeting the requirements for measurements. Compared to the interpolation method, our model achieves an error reduction of approximately 3 to 25 times, making it highly suitable for numerical processing in measurement tasks.

In practical applications, this model can provide a reliable solution for monitoring high-resolution temperature fields. Due to its universal design, the model has wider applicability and can adapt to monitoring requirements in various temperature fields. Despite significant progress in the current model design, challenges remain. In the current experiment, we still use a simple simulation model. Obtaining real experimental data to replace the simulation data currently used will be the focus of future research.

## Figures and Tables

**Table 1:** Related parameters of experimental data.

Scenarios	Laser Heating	Water Mixing	Heat Dissipating
Area Size	0.02 m × 0.02 m	0.03 m × 0.1 m	0.1 m × 0.2 m
Fine mesh	128×128		
Coarse mesh	4×4, 8×8, 16×16		
Sampling interval	0.01 s	0.001 s	0.01 s

**Table 2:** Performance comparison in laser heating scenarios (best scores in bold).

Coarse Mesh	Methods	MAPE	L2 Error	PSNR	SSIM
4×4	BI	3.073%	0.178	50.060	0.849
	BC	3.062%	0.177	**52.053**	**0.911**
	PI-Unet	46.650%	0.683	7.643	0.348
	DSC/MS	0.831%	0.096	41.437	0.766
	Modified DSC/MS	**0.422%**	**0.076**	45.377	0.729
8×8	BI	3.036%	0.176	59.000	0.975
	BC	3.037%	0.176	**60.767**	**0.976**
	PI-UNet	47.305%	0.689	7.456	0.185
	DSC/MS	0.626%	0.061	49.111	0.927
	Modified DSC/MS	**0.256%**	**0.076**	45.432	0.825
16×16	BI	3.031%	0.176	68.071	0.996
	BC	3.032%	0.176	**69.243**	**0.998**
	PI-UNet	45.615%	0.703	7.531	0.474
	DSC/MS	0.624%	0.081	44.223	0.959
	Modified DSC/MS	**0.116%**	**0.038**	55.708	0.994

**Table 3:** Performance comparison in water mixing scenarios (best scores in bold).

Coarse Mesh	Methods	MAPE	L2 Error	PSNR	SSIM
4×4	BI	18.402%	0.457	17.908	0.493
	BC	16.229%	0.433	18.773	0.424
	PI-UNet	**6.015%**	**0.299**	**23.362**	**0.550**
	DSC/MS	7.126%	0.346	20.770	0.499
	Modified DSC/MS	15.614%	0.461	15.778	0.247
8×8	BI	6.979%	0.299	24.177	0.678
	BC	5.610%	0.274	25.659	0.652
	PI-UNet	5.46%	0.268	25.277	0.678
	DSC/MS	3.393%	0.218	28.784	0.776
	Modified DSC/MS	**2.260%**	**0.174**	**32.550**	**0.769**
16×16	BI	2.903%	0.214	29.409	0.858
	BC	2.268%	0.193	31.023	0.857
	PI-UNet	5.402%	0.264	25.464	0.857
	DSC/MS	0.882%	0.122	38.588	0.774
	Modified DSC/MS	**0.127%**	**0.041**	**57.815**	**0.927**

**Table 4:** Performance comparison in heat dissipating scenarios (best scores in bold).

Coarse Mesh	Methods	MAPE	L2 Error	PSNR	SSIM
4×4	BI	123.444%	0.745	17.715	0.412
	BC	116.672%	**0.729**	**18.290**	0.442
	PI-UNet	57.695%	0.739	17.698	**0.471**
	DSC/MS	**36.369%**	0.733	17.374	0.353
	Modified DSC/MS	46.276%	0.751	16.995	0.268
8×8	BI	82.514%	0.630	21.473	**0.679**
	BC	77.621%	0.599	22.837	0.644
	PI-UNet	17.011%	0.454	26.158	0.659
	DSC/MS	15.570%	0.479	24.713	0.669
	Modified DSC/MS	**12.050%**	**0.436**	**26.409**	0.677
16×16	BI	63.203%	0.530	27.245	**0.879**
	BC	60.446%	0.501	29.596	0.877
	PI-UNet	9.876%	0.320	32.237	0.785
	DSC/MS	2.670%	**0.249**	**36.150**	0.863
	Modified DSC/MS	**2.621%**	0.268	35.208	0.870

**Table 5:** The influence of physical loss on prediction under different scenarios.

Scenarios	Coarse Mesh	Methods	MAPE	L2 Error	PSNR	SSIM
Laser Heating	4×4	Modified DSC/MS	0.422%	0.076	45.377	**0.729**
		Modified PI-DSC/MS	**0.406%**	**0.074**	**45.809**	0.719
	8×8	Modified DSC/MS	0.256%	0.076	45.432	0.825
		Modified PI-DSC/MS	**0.076%**	**0.031**	**60.891**	**0.982**
	16×16	Modified DSC/MS	0.116%	0.038	55.708	0.994
		Modified PI-DSC/MS	**0.027%**	**0.026**	**69.040**	**0.999**
Water Mixing	4×4	Modified DSC/MS	15.614%	0.461	15.778	0.247
		Modified PI-DSC/MS	**2.034%**	**0.192**	**30.938**	**0.618**
	8×8	Modified DSC/MS	2.260%	0.174	32.550	**0.769**
		Modified PI-DSC/MS	**0.300%**	**0.071**	**48.258**	0.766
	16×16	Modified DSC/MS	0.127%	0.041	57.815	0.927
		Modified PI-DSC/MS	**0.109%**	**0.039**	**58.611**	**0.932**
Heat Dissipating	4×4	Modified DSC/MS	46.276%	**0.751**	**16.995**	0.268
		Modified PI-DSC/MS	**32.296%**	0.758	16.775	**0.349**
	8×8	Modified DSC/MS	12.050%	0.436	26.409	**0.677**
		Modified PI-DSC/MS	**10.337%**	**0.425**	**26.787**	0.640
	16×16	Modified DSC/MS	2.621%	0.268	35.208	**0.870**
		Modified PI-DSC/MS	**2.262%**	**0.241**	**36.213**	0.856

**Table 6:** The influence of sampling quantity on the algorithm (taking the LR grid of 16×16 as an example).

Sample Quantity	Methods	MAPE	L2 Error	PSNR	SSIM
200	Modified DSC/MS	3.463%	0.256	35.657	0.837
	Modified PI-DSC/MS	4.587%	0.267	34.912	0.828
1000	Modified DSC/MS	2.541%	0.242	36.588	**0.877**
	Modified PI-DSC/MS	2.909%	**0.241**	**36.674**	0.856
2000	Modified DSC/MS	2.621%	0.268	35.208	0.870
	Modified PI-DSC/MS	**2.262%**	**0.241**	36.213	0.856

## Data Availability

The raw data supporting the conclusions of this article will be made available by the authors on request.

## Abbreviations

The following abbreviations are used in this manuscript:

CNN	Convolutional neural networks
DSC	Downsampled skip connection
HR	High resolution
LR	Low resolution
MS	Multi-scale
PINNs	Physics-informed neural networks
SR	Super-resolution

## References

[1] Gebhardt J., Sosale G., Dasgupta S. (2020). Non-invasive temperature measurement of turbulent flows of aqueous solutions and gases in pipes. Tm-Tech. Mess..

[2] Wang G., Sheng K., Wang Y., Ding G., Xie D. (2024). Thermal hydraulic performance of tree-like microchannel heat sink with high branching level based on the improved Murray’s law. Int. J. Heat Mass Transf..

[3] Doty J., Yerkes K., Byrd L., Murthy J., Alleyne A., Wolff M., Heister S., Fisher T. (2017). Dynamic thermal management for aerospace technology: Review and outlook. J. Thermophys. Heat Transf..

[4] Childs P.R., Greenwood J., Long C. (2000). Review of temperature measurement. Rev. Sci. Instrum..

[5] Upschulte B., Miller M., Allen M. (2000). Diode laser sensor for gasdynamic measurements in a model scramjet combustor. AIAA J..

[6] Griffiths A.D., Houwing A.F.P. (2005). Diode laser absorption spectroscopy of water vapor in a scramjet combustor. Appl. Opt..

[7] Krizhevsky A., Sutskever I., Hinton G.E. (2012). Imagenet classification with deep convolutional neural networks. Adv. Neural Inf. Process. Syst..

[8] Dong C., Loy C.C., He K., Tang X. (2015). Image super-resolution using deep convolutional networks. IEEE Trans. Pattern Anal. Mach. Intell..

[9] Keys R. (1981). Cubic convolution interpolation for digital image processing. IEEE Trans. Acoust. Speech Signal Process..

[10] Dai M., Xiao G., Fiondella L., Shao M., Zhang Y.S. (2021). Deep learning-enabled resolution-enhancement in mini-and regular microscopy for biomedical imaging. Sens. Actuators A Phys..

[11] Tang Y., Zhang J., Yue M., Qu Z., Wang X., Gui Y., Feng X. (2021). Deep learning-based super-resolution images for synchronous measurement of temperature and deformation at elevated temperature. Optik.

[12] Kong C., Chang J.T., Li Y.F., Chen R.Y. (2020). Deep learning methods for super-resolution reconstruction of temperature fields in a supersonic combustor. AIP Adv..

[13] Fukami K., Fukagata K., Taira K. (2019). Super-resolution reconstruction of turbulent flows with machine learning. J. Fluid Mech..

[14] Deng Z., He C., Liu Y., Kim K.C. (2019). Super-resolution reconstruction of turbulent velocity fields using a generative adversarial network-based artificial intelligence framework. Phys. Fluids.

[15] Cuomo S., Di Cola V.S., Giampaolo F., Rozza G., Raissi M., Piccialli F. (2022). Scientific machine learning through physics–informed neural networks: Where we are and what’s next. J. Sci. Comput..

[16] Cai S., Mao Z., Wang Z., Yin M., Karniadakis G.E. (2021). Physics-informed neural networks (PINNs) for fluid mechanics: A review. Acta Mech. Sin..

[17] Cai S., Wang Z., Wang S., Perdikaris P., Karniadakis G.E. (2021). Physics-informed neural networks for heat transfer problems. J. Heat Transf..

[18] Esmaeilzadeh S., Azizzadenesheli K., Kashinath K., Mustafa M., Tchelepi H.A., Marcus P., Prabhat M., Anandkumar A. (2020). Meshfreeflownet: A physics-constrained deep continuous space-time super-resolution framework. Proceedings of the SC20: International Conference for High Performance Computing, Networking, Storage and Analysis.

[19] Arora R. (2022). PhySRNet: Physics informed super-resolution network for application in computational solid mechanics. Proceedings of the 2022 IEEE/ACM International Workshop on Artificial Intelligence and Machine Learning for Scientific Applications (AI4S).

[20] Sarkar R.K., Majumdar R., Jadhav V., Sakhinana S.S., Runkana V. (2023). Redefining Super-Resolution: Fine-mesh PDE predictions without classical simulations. arXiv.

[21] COMSOL Laser Heating of a Silicon Wafer. https://www.comsol.com/model/laser-heating-of-a-silicon-wafer-13835.

[22] Nguyen T.K., Ba T.N., Ngoc P.B., Embong A.M., Nhu N.N.T., Xuan A.N.S., Trung N.H., Kadir N.A.A., Nguyen T.A., Pham L.H.H.P. (2021). Simulation of shell and tube heat exchanger using COMSOL software. Proceedings of the IOP Conference Series: Earth and Environmental Science.

[23] COMSOL Bimetallic Strip in Airflow. https://comsol.com/model/bimetallic-strip-in-airflow-74251.

[24] Ngiam J., Chen Z., Chia D., Koh P., Le Q., Ng A. (2010). Tiled convolutional neural networks. Adv. Neural Inf. Process. Syst..

[25] He K., Zhang X., Ren S., Sun J. Deep residual learning for image recognition. Proceedings of the IEEE Conference on Computer Vision and Pattern Recognition.

[26] Du X., Qu X., He Y., Guo D. (2018). Single image super-resolution based on multi-scale competitive convolutional neural network. Sensors.

